# Orai1–STIM1 Regulates Increased Ca^2+^ Mobilization, Leading to Contractile Duchenne Muscular Dystrophy Phenotypes in Patient-Derived Induced Pluripotent Stem Cells

**DOI:** 10.3390/biomedicines9111589

**Published:** 2021-10-31

**Authors:** Tomoya Uchimura, Hidetoshi Sakurai

**Affiliations:** 1 Center for iPSC Cell Research and Application (CiRA), Kyoto University, 53 Shogoin-Kawahara-cho, Sakyo-ku, Kyoto 606-8507, Japan; 2 Takeda-CiRA Joint Program, Fujisawa 251-8555, Japan

**Keywords:** iPSC, skeletal muscle, Ca^2+^ overload, store-operated Ca^2+^ channel, STIM1-Orai1

## Abstract

Ca^2+^ overload is one of the factors leading to Duchenne muscular dystrophy (DMD) pathogenesis. However, the molecular targets of dystrophin deficiency-dependent Ca^2+^ overload and the correlation between Ca^2+^ overload and contractile DMD phenotypes in in vitro human models remain largely elusive. In this study, we utilized DMD patient-derived induced pluripotent stem cells (iPSCs) to differentiate myotubes using doxycycline-inducible MyoD overexpression, and searched for a target molecule that mediates dystrophin deficiency-dependent Ca^2+^ overload using commercially available chemicals and siRNAs. We found that several store-operated Ca^2+^ channel (SOC) inhibitors effectively prevented Ca^2+^ overload and identified that STIM1–Orai1 is a molecular target of SOCs. These findings were further confirmed by demonstrating that STIM1–Orai1 inhibitors, CM4620, AnCoA4, and GSK797A, prevented Ca^2+^ overload in dystrophic myotubes. Finally, we evaluated CM4620, AnCoA4, and GSK7975A activities using a previously reported model recapitulating a muscle fatigue-like decline in contractile performance in DMD. All three chemicals ameliorated the decline in contractile performance, indicating that modulating STIM1–Orai1-mediated Ca^2+^ overload is effective in rescuing contractile phenotypes. In conclusion, SOCs are major contributors to dystrophin deficiency-dependent Ca^2+^ overload through STIM1–Orai1 as molecular mediators. Modulating STIM1–Orai1 activity was effective in ameliorating the decline in contractile performance in DMD.

## 1. Introduction

Duchenne muscular dystrophy (DMD) is a progressive muscular degeneration disease caused by the complete loss of dystrophin protein, eventually leading to ambulatory and respiratory deficiency, whose symptoms typically occur in early childhood [1]. Although several decades have passed since dystrophin was identified [2], standard glucocorticoid treatment is most routinely used [3,4]. The latest therapy includes micro-dystrophin gene delivery and myostatin inhibitors [5,6]. Moreover, exon skipping is considered a promising therapy for DMD [7]. However, current treatment options are still markedly limited. DMD pathogenesis progresses with muscle contractures [8], and early DMD can be evaluated by creatine kinase (CK) leakage [9]. While a loss of dystrophin is an initial trigger of DMD, mitochondrial dysfunction contributes to DMD pathogenesis, leading to reactive oxygen species (ROS) production, ATP depletion, organelle membrane permeabilization, and the release of factors that induce necrosis and inflammation [10,11,12,13,14,15,16]. Intracellular Ca^2+^ overload is also considered to regulate DMD pathogenesis. However, the association between dystrophin deficiency and pathogenesis progression remains unclear, and related treatment options targeting such mechanisms have not yet been developed.

Induced pluripotent stem cell (iPSC) technology has markedly advanced the field of regenerative medicine, including cell therapies and drug development, as well as basic science, facilitating the understanding of intractable disease pathogenesis [17]. It is important to establish a disease model representing disease clinical symptoms or molecular pathogenesis.

Ca^2+^ overload has been widely accepted as a candidate regulating DMD pathogenesis [18]. However, whether modulating Ca^2+^ overload has therapeutic effects for DMD in clinical settings remains largely unknown, due to the limited number of compounds available for clinical use or under development. We have previously reported a Ca^2+^ overload phenotype in myotubes differentiated from DMD patient-derived iPSCs [19]. Muscle contraction is tightly regulated by Ca^2+^ mobilization, and the store-operated Ca^2+^ channel (SOC) is one of the channels maintaining the intracellular Ca^2+^ concentration [20]. SOCs are activated by sarcoplasmic reticulum (SR) intraluminal Ca^2+^ ([Ca^2+^]_SR_) reduction, and SOCs normally provide Ca^2+^ to refill the SR and maintain homeostasis [21]. STIM1 and Orai1 are known as SOCs [21]. STIM1 localizes to the SR membrane and functions as a Ca^2+^ sensor in the SR and Orai1 localizes in the transverse tubule membrane and functions as a Ca^2+^ channel [22,23,24]. Orai1-dependent Ca^2+^ mobilization regulates skeletal muscle growth and fatigue as well as exacerbating Ca^2+^ entry in dystrophic muscles in *mdx* mice [23,25]. In contrast, STIM1 also regulates Ca^2+^ homeostasis during skeletal muscle development and function [26]. In addition, it also regulates myogenesis in human myotubes by interacting with transient receptor potential channel (TRPC) 1 and 4 [27]. Thus, STIM1–Orai1-regulated Ca^2+^ mobilization is important for muscle development and function [22]. Moreover, STIM1–Orai1 is involved in muscular pathology in DMD in a mouse model [28]. However, whether STIM1–Orai1-regulated Ca^2+^ mobilization contributes to Ca^2+^ overload as well as functional phenotypes in an in vitro human model remains unknown.

In this study, we identified that SOCs are potent regulators of dystrophin deficiency-mediated Ca^2+^ overload in myotubes differentiated from DMD patient-derived iPSCs using small-scale chemical screening. siRNA-mediated gene knockdown identified STIM1 and Orai1 as potential targets responsible for SOC-mediated Ca^2+^ overload in skeletal muscle cells. In addition, this result was further confirmed using STIM1 and Orai1 inhibitors, which prevented Ca^2+^ overload in dystrophic myotubes. Finally, to demonstrate how modulation of Ca^2+^ overload is linked to the functional rescue of dystrophic phenotypes, we utilized a recently reported disease model to evaluate the contractile performance decline in DMD. Orai1 and STIM1 inhibitor administration successfully rescued the decline in contractile performance. Thus, this study indicated that modulating STIM1–Orai1-regulated Ca^2+^ overload improves functional phenotypes and their potential for use in therapeutic purposes.

## 2. Materials and Methods

### 2.1. Ethical Approval

This study was approved by the Ethics Committee of the Graduate School of Medicine, Kyoto University and Kyoto University Hospital (approval number #R0091 and #G259, 5 September 2011), and Takeda Pharmaceutical Company Ltd. (GEN-00000040-019, 14 March 2019) and conducted according to the guidelines of the Declaration of Helsinki. All patient information was kept confidential, and written informed consent was obtained from all patients.

### 2.2. iPSC Lines

The Δ44 DMD-iPSCs established from skin fibroblasts of a DMD patient (exon ∆44 deletion, male 3 years old) and its isogenic control line, ∆44 DMD-ctrl-iPSCs as described previously [19,29], were used for the experiments.

### 2.3. Plate Preparation

For the preparation of laminin-coated plates, 1.5 or 8 mL of Easy iMatrix-511 silk (Nippi; Tokyo, Japan) were added to each well of the six-well plates or a 10-cm dish, respectively, and incubated overnight at 4 °C. The coated plates were stored at 4 °C for up to 2 weeks and equilibrated at 24 °C for at least 30 min prior to use. StemFit AK02N (StemFit; Reprocell; Beltsville, MD, USA) (750 µL or 3 mL) was added to wash the plates and aspirated. For the Matrigel-coated plates, all tubes, pipette tips, and reagents were pre-chilled in the fridge or on ice. Matrigel (BD; Franklin Lakes, NJ, USA) was prepared at 1:100 dilution with pre-chilled media without any additives. An appropriate volume of diluted Matrigel was dispensed into each well of the 6- or 96-well plates, which were incubated overnight at 4 °C and stored at 4 °C for a few weeks until use. For the gel culture, 2 mL or 10 µL of 0.5 mg/mL collagen solution (Nippi) were dispensed into a well of 6-well plates (BD) or µPlate Angiogenesis 96 plate (Ibidi; Planegg, Germany) and solidified in an incubator at 37 °C overnight. The plates were then coated with Matrigel.

### 2.4. Generation of the iPSC Line Stably Expressing Tet-Inducible MyoD1

iPSC lines stably expressing Tet-inducible MyoD1 were generated as described previously [30]. Briefly, the iPSCs were dissociated into single cells using Accutase (Nacalai; Kyoto, Japan), and 1.0 × 10^6^ cells were resuspended in Opti-MEM (Invitrogen; Carlsbad, CA, USA). Doxycycline (Dox)-inducible MyoD1-expressing *piggyBac* vector, Tet-MyoD [31], was co-electroporated with the *piggyBac* transposase vector PBaseII using NEPA 21 at 125 V for 5 ms (Nepagene; Chiba, Japan). After selecting the successfully differentiated clones from puromycin-resistant colonies, the cells were established and used for experiments.

### 2.5. Feeder-Free iPSC Culture

The iPSCs were cultured on laminin-coated plates in StemFit AK02N (StemFit; Reprocell; Beltsville, MD, USA) containing 100 µg/mL G418 or 0.5 µg/mL puromycin, depending on the antibiotic resistance of each line. The cells were passaged every seven days using Accutase [32] and seeded on laminin-coated plates in the presence of 10 µM Y-27632 (Nacalai) at 1.5 × 10^4^ cells/well in 6-well plates or 10-cm dishes for the first two days after plating. At 48 h after passaging, Y-27632 was removed and replaced with StemFit containing a suitable antibiotic. The medium was changed at least every other day.

### 2.6. Skeletal Muscle Differentiation by the Standard Replating Method

The cells were re-plated as described previously [30]. Briefly, iPSCs were treated with Accutase and plated on Matrigel-coated plates in StemFit+10 µM Y-27632 at 3.0 × 10^4^ cells/cm^2^. After 24 and 48 h, the media were replaced with primate ES cell medium (PECM; Reprocell) and PECM+1 µg/mL Dox (Nacalai), respectively. The next day, the pre-differentiated iPSCs were treated with Accutase and re-plated into new Matrigel-coated microplates in 5% knockout serum replacement (KSR; ThermoFisher; Waltham, MA, USA) in αMEM (Nacalai)+Y-27632+1 µg/mL Dox. The medium was replaced every other day with fresh medium. To induce further maturation of myotubes, the cells were cultured in 2% horse serum (HS; Sigma; St. Louis, MO, USA)/αMEM. The medium was replaced a few times per week.

### 2.7. Skeletal Muscle Differentiation by the Modified Replating Method

iPSCs were treated with Accutase and plated on Matrigel-coated plates in StemFit+Y at 3.0 × 10^4^ cells/cm^2^. After 24 and 48 h, the media was replaced with PECM and PECM+0.3 µg/mL Dox, respectively. After an additional 48 h, the pre-differentiated iPSCs were treated with Accutase and re-plated into new Matrigel-coated microplates or hydrogels in 2% HS/αMEM supplemented with 200 µM 2-mercaptoethanol (Nacalai), 4.5 g/L glucose (Invitrogen), 10 µg/mL insulin (Wako; Richmond, VA, USA), SB431542 (Wako) and 3 µM Y-27632. After two days, the medium was replaced with 2% HS/αMEM+1 µg/mL Dox and changed every other day. To induce further maturation of myotubes, doxycycline was removed from the media and the cells were cultured in 2% HS/αMEM for 3 weeks. The medium was replaced every other day.

### 2.8. siRNA Transfection

For siRNA transfection, the cells were transfected with Lipofectamine RNAiMAX (ThermoFisher) by forward transfection with 1 *p*mol *Silencer* Select Negative Control No.1 siRNA or *Silencer* Select STIM1L and STIM1S (designed from a previously published article [33]) on day 10 of differentiation. On day 14 of differentiation, the cells were harvested by the scraper and pelleted by centrifuging at 3000 rpm for 5 min. The pellets were stored at −80 °C until use for RT-qPCR and Western blot analyses.

### 2.9. Immunocytochemistry

The cells were fixed with 2% paraformaldehyde (PFA)/PBS (Wako) and methanol (Wako), blocked with Blocking One (Nacalai) for 45 min, and subsequently incubated with primary antibodies diluted in 5% Blocking One/PBST (Wako) at 4 °C overnight. The cells were washed in PBS and incubated with secondary antibodies diluted in 5% Blocking One/PBST for 1 h at room temperature (24–26 °C). Then, 4′,6-diamidino-2-phenylindole (DAPI; Sigma) was used to counterstain the nuclei. The samples were visualized and photographed with a BZ-710X (Keyence; Osaka, Japan) or Opera Phenix System (PerkinElmer; Waltham, MA, USA). The primary antibodies used for this study were: mouse anti-myosin heavy-chain (pan-MHC) monoclonal (MF20; 1:500; R&D; Minneapolis, MN, USA), mouse anti-dystrophin (Rod domain) monoclonal (DYS1; 1:20; Leica; Buffalo Grove, IL, USA), rabbit anti-STIM1 monoclonal (D88E10; 1:800; Cell Signaling Technology; Danvers, MA, USA), and mouse anti-Orai1 monoclonal (G-2; 1:5; Santa Cruz; Dallas, TX, USA) antibodies. Alexa Fluor 488-conjugated anti-mouse/rabbit and Alexa Fluor 647-conjugated anti-mouse/rabbit antibodies (1:500, Invitrogen) were used as secondary antibodies.

### 2.10. RT-qPCR Analysis

Total RNA was isolated using the RNeasy Mini Kit (Qiagen; Hilden, Germany) according to the manufacturer’s protocol. The residual genomic DNA was digested and removed using DNase I (Qiagen). First-strand cDNA was generated from 100 ng of total RNA using a PrimeScript RT reagent kit (Takara; Kyoto, Japan). Quantitative PCR was performed using PowerUp SYBR Green (ThermoFisher) or TaqMan assay (Applied Biosystems; Foster City, CA, USA) and QuantStudio 7 Flex (Applied Biosystems). Ribosomal protein lateral stalk subunit P0 (*RPLP*) served as the reference gene for TaqMan assays, and TATA-binding protein (*TBP*) served as a reference gene for SYBR Green assays. The following TaqMan probes were used: *MYH1* (Hs00428600), *MYH2* (Hs00430042), *MYH3* (Hs01074230), *MYH4* (Hs00757977), *MYH7* (Hs0110632), *MYH8* (Hs00267293), *IL-1β* (Hs00174097), *TNFα* (Hs01113624), *IL6* (Hs00985639), and *RPLP0* (Hs99999902). The sequences for the primers used for SYBR Green are shown in the Supplementary Materials Table S1.

### 2.11. Western Blot Analysis

The cells were lysed using cOmplete lysis-M-buffer (Roche; Basel, Switzerland) in the presence of a cOmplete Mini protease inhibitor cocktail (Roche). Protein concentrations were determined using a bicinchoninic acid (BCA) protein assay kit (Takara). For sodium dodecyl sulfate polyacrylamide gel electrophoresis (SDS–PAGE), 4 × Laemmli sample buffer and any KD mini-protean TGX precast gel with Mini-Protean Tetra Cell (Bio-Rad; Hercules, CA, USA) were used to load samples, except the DYS1 and RYR1 samples, which were loaded using 4 × LDS sample buffer, 10 × reducing agent and 3–8%NuPAGE, and Tris-acetate protein precast gels with Mini Gel Tank (ThermoFisher). Western blot analysis was performed using the iBind Flex Western Device (ThermoFisher), PVDF membrane (Bio-Rad), and the following primary antibodies: mouse anti-myosin heavy-chain (pan-MHC) monoclonal (MF20, 1:1000, R&D), mouse anti-dystrophin (Rod domain) monoclonal (DYS1, 1:20, Leica), and rabbit anti-TBP monoclonal (D5C9H, 1:1000, Cell Signaling) antibodies. A horseradish peroxidase (HRP)-conjugated rabbit anti-mouse antibody (GE; Chicago, IL, USA) was used as a secondary antibody, and ECL Select or Prime Western Blotting Detection Reagent (GE) were used for protein visualization.

### 2.12. Ca^2+^ Mobilization Assay

The Ca^2+^ mobilization assay was conducted as previously described [19]. Briefly, the cells were differentiated on Matrigel-coated 96-well plates (PerkinElmer) and loaded with Cal-520 AM, a fluorescent Ca^2+^ indicator (AAT Bioquest; Sunnyvale, CA, USA), in FluoroBrite Dulbecco’s modified Eagle’s medium (DMEM, Invitrogen) in the presence or absence of each chemical and dimethyl sulfoxide (DMSO) control and incubated at 37 °C for 1 h. The fluorescence was detected using an FDSS/µCELL system (Hamamatsu Photonics; Shizuoka, Japan). Electrical stimulation was applied at 15 V with a 50-ms interval and 0.2 Hz mono phase for 1 min after a 5-s resting phase. The fluorescence was measured at 480 nm excitation and 540 nm emission using an LED excitation light source and an electron-multiplying charge-coupled device (EMCCD) camera. Measurements for 96 wells were performed under uniform conditions at 37 °C with simultaneous stimulation and detection.

### 2.13. Total Ca^2+^ Content Measurement

Total releasable Ca^2+^ was determined according to a previously published protocol [34,35]. Briefly, the cells were differentiated on Matrigel-coated 96-well plates. On day 14, the cells were loaded with 4 µM fura-FF AM (AAT Bioquest) in Ringer’s solution for 30 min in an incubator at 37 °C and 5% CO_2_. Then, they were washed with Ca^2+^-free Ringer’s solution for 30 min in an incubator, and fresh Ca^2+^-free Ringer’s solution was added. The cells were excited at 340 and 380 nm, and the emission at 535 nm was captured using an EnVision (Perkin Elmer). A Ca^2+^ release cocktail containing 10 µM ionomycin (Wako), 30 µM CPA, and 100 µM EGTA in Ca^2+^-free Ringer’s solution was injected 5 s after the start of imaging. The total releasable Ca^2+^ content was determined by calculating the difference between the basal and peak fura-FF ratios.

### 2.14. Electrical Field Stimulation (EFS) and Motion Imaging Assays

The cells were differentiated on Matrigel-coated 6-well collagen gel plates and stimulated by an EFS system with C-Pace EP and 6-well C-Dish (IonOptics; Westwood, MA, USA) for differentiated myotube maturation and muscle cell training. A movie was recorded and analyzed using an SI8000 Motion Imaging System (SONY; Tokyo, Japan) equipped with an inverted fluorescent microscope (Eclipse Ti; Nikon; Tokyo, Japan) and a stage top incubator (Tokai Hit; Shizuoka, Japan) to maintain the cells under humidified conditions at 37 °C and 5% CO_2_. Six-well hydrogel or collagen gel plates with cells were cultured in an incubator equipped with a C-Dish connected to the C-Pace EP. An EFS was applied between 2 and 20 V with a 2-ms interval and 0.5 and 1 Hz. The movie was recorded for 270 frames at 27 f/s (equal to 10 s) and analyzed using the associated software.

### 2.15. Microscopy

The cultured cells were visualized under a BZ-X710 fluorescence microscope (Keyence) and an Opera Phenix High-Content Screening System (PerkinElmer). The images were acquired and analyzed using the associated software.

### 2.16. Statistics Analysis

All experiments were conducted independently at least in triplicate. Data are reported as the mean ± standard deviation (SD) and were analyzed using an unpaired *t*-test, Dunnett’s test, or one-way analysis of variance (ANOVA), followed by post-hoc Dunnett’s test using GraphPad Prism (Version 9.2.0; GraphPad Software; San Diego, CA, USA). Statistical significance was set at *p* < 0.05.

## 3. Results

### 3.1. Ca^2+^ Overload Condition

**Figure 1:**
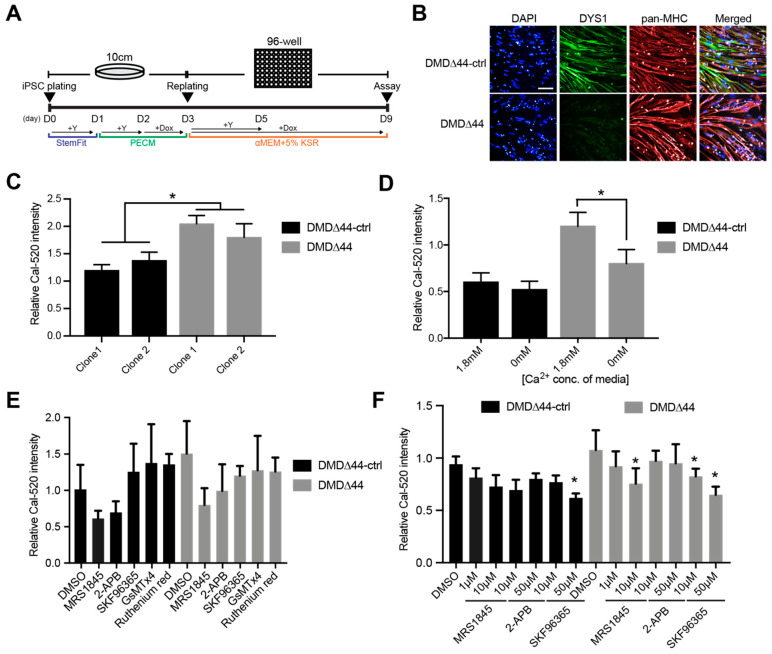
Store-operated Ca^2+^ channels (SOCs) regulate Ca^2+^ overload in dystrophic myotubes using induced pluripotent stem cells (iPSCs). (**A**) A schematic diagram of the standard replating method. The iPSCs (DMD∆44 and DMD∆44-ctrl) were myogenically pre-differentiated in primate ES cell medium (PECM) in the presence of doxycycline and re-plated on day 3 in the presence of Y-27632 and doxycycline in 5% knockout serum replacement (KSR) media. (**B**) Immunocytochemical analysis of DYS1 and pan-MHC in differentiated myotubes on day 9. Scale bar = 200 µm. (**C**–**F**) Relative Ca^2+^ concentration using DMD∆44-ctrl and DMD∆44 cell lines and (**D**) Ca^2+^-free media, (**E**) Ca^2+^ channel inhibitors, and (**F**) SOC inhibitors. * indicates *p* < 0.05. Y = Y-27632, Dox = doxycycline, Conc. = concentration.

In this study, we used two different plating methods: the standard replating method and the modified replating method [30,36], both of which were previously reported by us. Since the advantage of the standard method is that iPSCs can be differentiated into myotubes within 14 days without maturation (Figure 1A), which is beneficial for the initial study, we first confirmed whether the Ca^2+^ overload condition was recapitulated from dystrophic myotubes using the standard method. In the standard replating method, both DMD∆44-ctrl and DMD∆44 iPSCs were plated on a 10-cm dish and treated with doxycycline to induce MyoD-overexpression-mediated myogenic differentiation before the cells were replated on day 3. On day 3, cells were plated in a 96-well plate in the presence of rock inhibitor and doxycycline in 5% KSR medium and assayed on day 9 of myogenic differentiation. An isogenic control of DMD∆44, whose dystrophin expression was restored [29], was used as the control (Figure 1B), and successful myogenic differentiation was confirmed (Figure S1). Using two different clones of each DMD∆44-ctrl and DMD∆44, we conducted Ca^2+^ mobilization assays using a Ca^2+^ indicator, and Ca^2+^ overload conditions were observed in DMD∆44 myotubes (Figure 1C). Since SR and mitochondria store internal Ca^2+^ in muscles, we focused on identifying whether intracellular or extracellular space is the source of increased Ca^2+^. Accordingly, we performed Ca^2+^ mobilization assays using the media in the presence or absence of Ca^2+^. Using Ca^2+^-free media in the assay, we aimed to estimate the contribution of extracellular Ca^2+^ to the increased Ca^2+^. The Ca^2+^ concentration between the DMD∆44-ctrl cells grown in Ca^2+^- and Ca^2+^-free media was not significantly different, indicating that the influxed Ca^2+^ is mostly from the inside of cells, such as SR and mitochondria-related cells (Figure 1D). Hence, it can be expected that Ca^2+^ from the extracellular space is normally used to maintain the internal Ca^2+^ concentration. On the contrary, Ca^2+^ overload was significantly reduced between the DMD∆44-ctrl and DMD∆44 in Ca^2+^-free media, indicating that approximately 60% increased Ca^2+^ was from the extracellular space, and the rest was from the SR and mitochondria-related cells (Figure 1D). As SOC is activated by local and transient depletion of intracellular Ca^2+^ stores [37], we measured the total Ca^2+^ store content under resting conditions using the method previously established by Michelucci et al. [34]. We found that the total Ca^2+^ store content was lower in DMD∆44 myotubes than in DMD∆44-ctrl myotubes (Figure S2), indicating that a partial reduction in SR Ca^2+^ might be sufficient to activate SOC in dystrophic myotubes. Nevertheless, our results indicate that Ca^2+^ channels regulating Ca^2+^ influx from the extracellular space are also important in mediating normal Ca^2+^ mobilization in dystrophic muscle.

### 3.2. SOCs

Based on the previous results, we decided to investigate the extracellular space Ca^2+^-dependent Ca^2+^ overload because our results indicate that more than half of the increased Ca^2+^ was from the extracellular space. In addition, it is more feasible to deliver the drugs to Ca^2+^ channels located within the extracellular membrane than the SR located inside the cells. Since there are several types of Ca^2+^ channels, such as TRPC, voltage-gated Ca^2+^ channel (VGCC), Na^+^/Ca^2+^ exchanger, and SOC, we chemically screened the channel responsible for Ca^2+^ overload in the model using 10 µM solutions of commercially available compounds with different chemical activities and molecular targets (Table 1). We observed that multiple SOC-inhibiting chemicals, including MRS1845, 2-APB, and SKF96365, effectively inhibit Ca^2+^ overload (Figure 1E,F). On the contrary, most non-SOC inhibitors, such as GsMTx4 (TRPC1 and 6 inhibitor) [38] or ruthenium red (TRPV inhibitor, mitochondrial Ca^2+^ uptake inhibitor) [39], did not prevent the overload or activities were not repeated (Figure 1E). To further confirm the inhibitory activities of MRS1845, 2-APB, and SKF96365, we repeated a Ca^2+^ mobilization assay and confirmed the effects in a dose-dependent manner (Figure 1F). Although it has been reported that the inhibitory action of 2-APB depends on the dose in non-muscle cells [21,40], it seems to have inhibitory effects on iPSC-derived skeletal myotubes in our study. These data indicate that SOCs could be a potent target for modulating Ca^2+^ overload in dystrophic myotubes.

### 3.3. Orai1 and STIM1 Inhibitors

**Figure 2:**
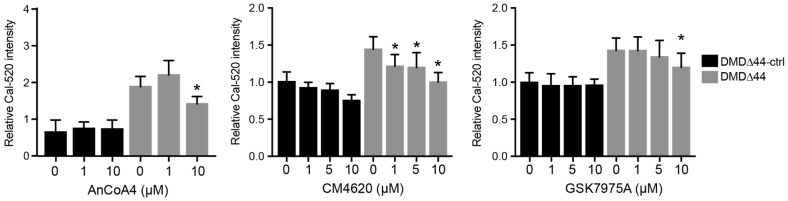
STIM1–Orai1 regulated Ca^2+^ overload in dystrophic myotubes. The Ca^2+^ peak level using Orai1/STIM1 inhibitors. * indicates *p* < 0.05.

Next, we aimed to identify the molecular targets of SOCs regulating Ca^2+^ overload in dystrophic myotubes. STIM1 and Orai1 are well-known SOCs, and the interaction between STIM1 and Orai1 regulates intracellular Ca^2+^ levels [21]. Normally, STIM1 and Orai1 are localized in the SR and transverse the tubule membrane, respectively [23,24,41]. Upon the reduction of Ca^2+^ levels in SR, STIM1 relocates to the plasma membrane–SR junction to bind to Orai1, triggering Orai1 activation. Thus, the prevention of STIM1 or Orai1 binding reduces Orai1-induced Ca^2+^ influx into the cytosol. Therefore, we studied whether Orai1 and STIM1 inhibitors also inhibit Ca^2+^ overload. First, we checked the mRNA expression levels of *Orai1* and *STIM1* during myogenic differentiation and found that both mRNA expression levels increased (Figure S1). In addition, there were no changes in *Orai1* and *STIM* mRNA expression levels between DMD∆44-ctrl and DMD∆44. Next, we utilized three different Orai1–STIM1 inhibitors, AnCoA4 [42], CM4620 [43], and GSK7975A, and evaluated the activities of each chemical in the Ca^2+^ mobilization assay. As expected, all three chemicals inhibited Ca^2+^ overload (Figure 2). Among the three chemicals, CM4620 is likely to be the most prominent, inhibiting Ca^2+^ overload even at 1 µM, as well as in a dose-dependent manner. These data indicate that STIM1–Orai1 is a molecular target that modulates Ca^2+^ overload in dystrophic myotubes.

### 3.4. Re-Evaluation of the Role of Orai1 and STIM1 in Matured Myotubes

**Figure 3:**
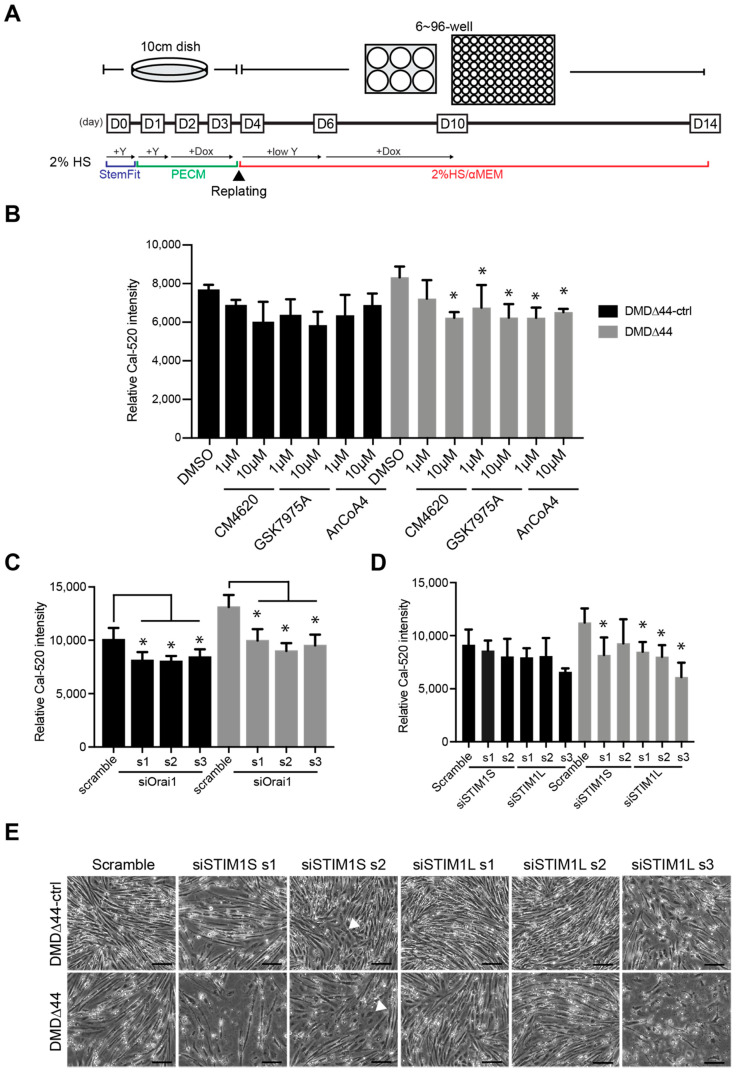
STIM1L–Orai1-regulated Ca^2+^ overload in dystrophic myotubes. (**A**) Schematic diagram of the modified replating method. Induced pluripotent stem cells (iPSCs; DMD∆44 and DMD∆44-ctrl) were myogenically pre-differentiated in primate ES cell medium (PECM) in the presence of doxycycline and re-plated on day 4 in the presence of low concentrations of Y-27632 and 2% horse serum media. (**B**–**D**) Relative Ca^2+^ peak level using Orai1/STIM inhibitors, siRNAs targeting *Orai1*, and siRNAs targeting *STIM1L*. (**E**) Bright field images of differentiated myotubes treated with siRNAs targeting *STIM1S* and *STIM1L* on day 14. Scale bar = 200 µM. Arrow heads indicate cells with abnormal differentiation upon siRNA treatment. * indicates *p* < 0.05.

As discussed previously, we recently reported a modified replating method to obtain mature myotubes from hiPSCs [36]. Although Ca^2+^ overload has already been recapitulated in the modified method, whether Orai1 and STIM1 regulate Ca^2+^ overload in dystrophic myotubes remains unknown. Thus, we differentiated myotubes using DMD∆44-ctrl and DMD∆44 iPSCs using the modified method (Figure 3A) and re-evaluated CM4620, AnCoA4, and GSK7975A activities. In the modified replating method, similar to the standard method, the iPSCs were pre-plated and treated with doxycycline for 48 h before replating. On day 4 of culture, cells were replated in the presence of a rock inhibitor without doxycycline. Doxycycline was administered on day 6 to induce MyoD-overexpression-mediated myogenic differentiation in 2% horse serum media for 4 days. From day 10, the medium was switched to doxycycline-free media for an additional 4 days, and the cells were assayed on day 14. As observed from the standard method, we also confirmed that all three chemicals were effective in preventing Ca^2+^ overload in DMD∆44 myotubes by the modified method (Figure 3B).

Next, we performed siRNA-mediated *Orai1* and *STIM1* knockdown using the modified method. *Orai1* mRNA knockdown was more than 90% efficient and significant reductions in increased Ca^2+^ levels were observed (Figure 3C and Figure S3). On the contrary, since *STIM1* has two isoforms, *STIM1S* and *STIM1L* [27], we designed siRNAs for *STIM1S* and *STIM1L* according to a previously published report [27] and conducted a Ca^2+^ mobilization assay. Interestingly, siRNAs targeting *STIM1L* were only effective against Ca^2+^ overload (Figure 3D and Figure S3). Although set 3 of siSTIM1L was toxic to the cells, as seen in the bright field image (Figure 3E), at least sets 1 and 2 did not cause any cell death or impaired myogenic differentiation (Figure 3E). In addition, *STIM1S* knockdown using both sets of siRNAs seemed to be toxic (Figure 3E), which may contribute to the slightly reduced Ca^2+^ level. Lastly, Orai1 and STIM1L mRNA and protein expression levels did not significantly change between DMD∆44-ctrl and DMD∆44, suggesting that dystrophin may not regulate their expression (Figure S3).

### 3.5. Effects of Orai1–STIM1 Inhibitors in the Muscle Training Model

**Figure 4:**
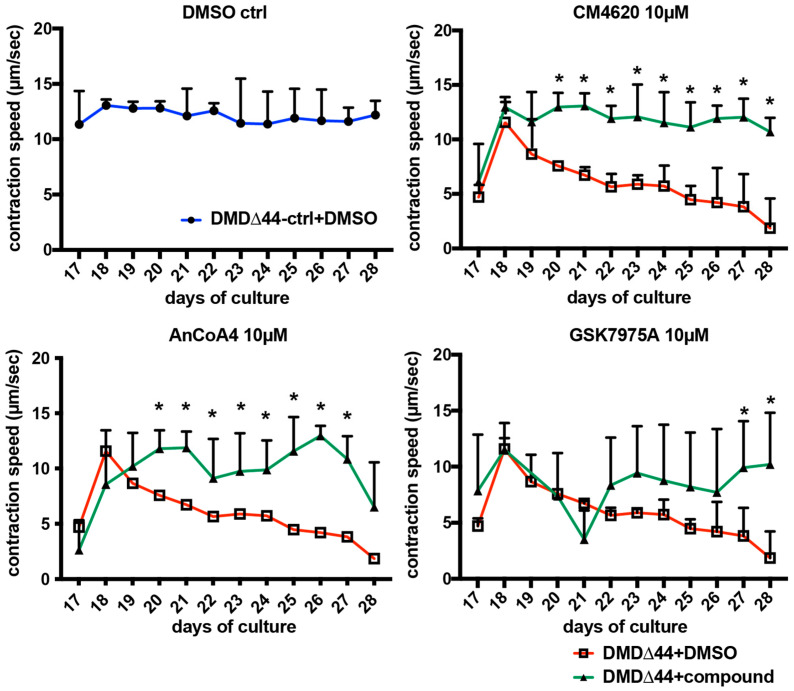
Amelioration of the declines in contractile performance upon administration of Orai1/STIM1 inhibitors in the long-term training model. Quantitative time-course analyses of the contraction velocity upon administration of Orai1/STIM1 inhibitors using DMD∆44-ctrl and DMD∆44. Data represent the mean ± standard deviation (SD) and were analyzed using an unpaired *t*-test from at least three biological replicates. * indicates *p* < 0.05.

As we identified that Orai1–STIM1 SOCs regulate dystrophin deficiency-dependent Ca^2+^ overload in our model, we investigated the effects of modulating Orai1–SIIM1-mediated Ca^2+^ overload on contractile performance. Recently, we reported a recapitulation of fatigue-like decline in contractile performance in DMD using an (EFS)-mediated muscle training model [36]. However, the interaction between Orai1–STIM1-regulated Ca^2+^ overload and contractile phenotypes remains unknown. To address this question, we evaluated the activities of CM4620, AnCoA4, and GSK7975A in this training model. In the DMSO control of DMD∆44-ctrl, the contractile performance was maintained for two weeks (Figure 4). On the contrary, in the DMSO control of DMD∆44, the contractile performance gradually declined, representing a muscle fatigue-like phenotype (Figure 4). Upon administration of the Orai1–STIM1 inhibitors, contractile phenotypes were ameliorated, indicating the activities of intervention by modulating Ca^2+^ mobilization via inhibition of Orai1 and STIM1 activities (Figure 4). Consistent with the Ca^2+^ mobilization assay, CM4620 was the most potent in rescuing the contractile performance. These data suggest correlations between Ca^2+^ mobilization and muscle performance phenotypes in dystrophic myotubes, and modulation of Orai1–STIM1 activity may be a potent target to ameliorate the contractile phenotypes in DMD.

## 4. Discussion

Ca^2+^ overload is a dystrophic phenotype that contributes to DMD pathogenesis [18]. While it has been proposed that Ca^2+^ overload triggers initial molecular pathogenesis, in turn leading to functional phenotypes, such as contractile dysfunction or muscle fiber degeneration, whether Ca^2+^ overload genuinely causes pathological changes in humans remains elusive. Skeletal muscle depends on an increased intracellular Ca^2+^ concentration during contraction. In general, Ca^2+^ concentrations in the extracellular space (2–4 mM), intracellular space (~100 nM), and inside the SR (~0.4 mM) were precisely maintained under normal conditions [44]. Ca^2+^ overload is defined as a condition where the intracellular Ca^2+^ concentration is abnormally higher than normal, leading to abnormally activated Ca^2+^ signaling. In turn, it is expected that abnormally activated Ca^2+^ signaling activates calpain signaling or proteolysis, ultimately leading to muscle weakness and degeneration [45]. In contrast to clinical or human models, the correlation between Ca^2+^ overload and muscle function in DMD has been well studied in animal models. Tutdibi et al. reported that ion channel blockers reduce Ca^2+^ entry into muscle fibers in *mdx* mice [46]. Milay et al. showed that TRPC-mediated Ca^2+^ influx is sufficient to induce DMD in *mdx* mice [13]. Bellinger et al. demonstrated that hypernitrosylated ryanodine receptors in *mdx* mice are leaky in handling Ca^2+^ [47]. Moreover, Rycal, an RYR Ca^2+^ release channel stabilizer, improves muscle fiber function in *mdx* mice, and Rycal ARM210/S48168 has been used in a clinical stage program [48]. However, although Ca^2+^ overload conditions have been recapitulated in vitro using human cells [19,36], whether Ca^2+^ overload conditions lead to the functional phenotype in human models remains unclear. Thus, a disease model that recapitulates functional DMD phenotypes is required for functional analysis, evaluation of potential compounds, and molecular pathogenesis studies.

This study aimed to reveal the molecular target of how dystrophin deficiency causes Ca^2+^ overload and correlations between Ca^2+^ overload and functional activities using myotubes differentiated from patient-derived iPSCs using a recently reported method [36]. Initial assays indicated that exacerbated Ca^2+^ could originate from both intracellular and extracellular space. Inside the cells, the SR–mitochondria axis contributes to increased Ca^2+^ levels in DMD. Upon cellular stimulation, robust Ca^2+^ is released from the SR to the cytoplasm. Thus, if the released Ca^2+^ amount from the SR is abnormally increased, it could contribute to Ca^2+^ overload. On the contrary, mitochondrial Ca^2+^ uptake may also contribute to Ca^2+^ overload. Normally, the Ca^2+^ concentration in the mitochondria is ~100 nM. However, it would reach ~100 µM upon Ca^2+^ signaling activation through mitochondrial Ca^2^ uptake [39,49]. Mitochondria function to buffer intracellular Ca^2+^. Dubinin et al. reported decreased mitochondrial Ca^2+^ uptake in *mdx* mice, indicating that mitochondria could also contribute to Ca^2+^ overload in DMD [14,50]. Since whether both SR and mitochondria contribute to Ca^2+^ overload in our model remains unknown, it would be interesting to further investigate this in future studies. However, Michelucci et al. reported a correlation between reduced Ca^2+^ content in the SR and increased Ca^2+^ influx through SOCs [34,35]. An unexpected finding of this study was the low basal level of SR Ca^2+^ content in dystrophic myotubes compared to that in control myotubes upon ionomycin application. While the molecular mechanism remains unclear, there are a few possible explanations. First, the responses of myotubes to ionomycin and EFS are different. Ionomycin is a calcium ionophore that facilitates Ca^2+^ transport across the plasma membrane, leading to Ca^2+^ release from intracellular stores [51]. On the contrary, EFS leads to membrane depolarization followed by excitation–contraction coupling [52], which may lead to less Ca^2+^ depletion in the SR compared to ionomycin. Thus, it will be important for future studies to assess the details of SR Ca^2+^ homeostasis in dystrophic myotubes under EFS. Second, correlated with this result, Robin et al. reported that the total SR Ca^2+^ content was reduced in *mdx* fibers compared to that in control fibers [53]. They also reported that a high sarcolemmal Ca^2+^ influx and SR leak contribute to high cytosolic Ca^2+^ levels in DMD. Thus, a high SR leak in DMD might explain the lower total SR Ca^2+^ store content in dystrophic myotubes than that in control myotubes reported in this study. Abnormal Ca^2+^ homeostasis in dystrophic myotubes may increase Ca^2+^ influx from the extracellular space as well as Ca^2+^ fluxes from the SR upon EFS. If this is a specific phenotype of dystrophic myotubes, further investigation of how dystrophin regulates the Ca^2+^ content in the SR and SOC activities, along with the reduction of Ca^2+^ in the SR could be a key to identify the molecular function of dystrophin in DMD.

In this study, we focused on the extracellular space because we targeted the development of a channel blocker that does not require the compounds to pass through the cell membrane. Using commercially available Ca^2+^ inhibitors/modulators, we identified that SOCs were the most attractive targets for regulating dystrophin deficiency-mediated Ca^2+^ overload. The role of SOCs in abnormal Ca^2+^ handling in DMD has been investigated, and targeting of SOCs to restore Ca^2+^ homeostasis for DMD therapies has been discussed previously [18,54]. While Orai1 and STIM1 are major components of SOCs, other Ca^2+^ channels, such as TRPC1, TRPV, and TRPM7, also contribute to SOC activity [55,56]. In this study, siRNA-mediated knockdown experiments revealed that Orai1 and STIM1 are the most prominent candidates for SOCs regulating Ca^2+^ overload in DMD. One of the limitations of our study is that ruthenium red inhibits TRPV activity and mitochondrial Ca^2+^ uptake [57,58]. In a previous study, we utilized ruthenium red as a TRPV inhibitor [19]. On the contrary, ruthenium red also inhibited mitochondrial Ca^2+^ uptake, increasing the cytoplasmic Ca^2+^ levels. Thus, ruthenium red treatment could possibly decrease the cytoplasmic Ca^2+^ levels via TRPV, increase cytoplasmic Ca^2+^ levels via mitochondrial Ca^2+^ uptake, or cause no change. While it remains elusive what targets the primary activity of ruthenium red in our model, further investigations of mitochondrial Ca^2+^ uptake would be interesting in future studies.

Correlations between Orai1–STIM1 and DMD have been previously shown in vivo. Zhao et al. found that upregulated Orai1-induced Ca^2+^ overload contributes to disrupted Ca^2+^ homeostasis in *mdx* muscles [25]. Wei-LaPierre et al. demonstrated an important physiological role of Orai1 activity in the muscles of mice, including fatigue [23]. Furthermore, Goonasekera et al. revealed that STIM1–Orai1-regulated Ca^2+^ mobilization induces a muscular dystrophy-like pathology in STIM1 transgenic, dominant-negative Orai1 mutant, and *mdx* mice [28]. These data indicate that there is a strong correlation between STIM1–Orai1 activities and DMD pathogenesis at the animal model level; however, whether this also occurs in humans remains unclear. Our study was consistent with previous in vivo studies, and supports the hypothesis that STIM1–Orai1 is a potential candidate for therapeutic purposes. In addition, our study also identified that STIM1L, an actin-binding splice variant of STIM, regulates Ca^2+^ overload without affecting myogenic differentiation [33]. Both STIM1S and STIM1L regulate Ca^2+^ homeostasis in different roles [33,59,60]. However, which variant or both contribute to DMD pathogenesis remain unclear. Our study indicated that Orai1–STIM1L is a potent target to prevent DMD pathogenesis.

Lastly, to assess the effects of modulating STIM1–Orai1 activities on the functional phenotype, we performed a functional analysis using a previously reported model [36]. In this model, a muscle fatigue-like decline in contractile performance was observed in dystrophic myotubes, and different Orai1–STIM1 inhibitors, CM4620 [43], AnCoA4 [42], and GSK7975A, were administered to determine whether modulating STIM1–Orai1 activities are effective in rescuing functional phenotypes. Although the preventive effects varied among the three different chemicals, all of them showed positive effects on functional rescue. Among the three chemicals, CM4620 is currently under clinical trial in patients with acute pancreatitis [43]. While whether CM4620 is also effective in ameliorating the functional DMD phenotypes in patients remains unclear, a further investigation to verify its effectiveness on DMD would be anticipated. In addition, it is important to characterize the differences between STIM1–Orai1 modulators and Rycal [47]. While Rycal targets Ca^2+^ leakage from the SR via RYR inside the cells, STIM1–Orai1 modulators target Ca^2+^ channels on the cell membrane and Ca^2+^ influx from the extracellular space. Rycal inhibits RYR1 S-nitrosylation-induced SR Ca^2+^ leakage [47], which could be caused by pathological changes during DMD progression. Therefore, it is also possible that both SR and extracellular space-derived Ca^2+^ influx contribute to DMD pathogenesis. In addition, Ca^2+^ leakage from the SR via RYR may also lead to hyperactivated STIM1–Orai1. Nevertheless, further investigation is necessary to determine the molecular pathology of Ca^2+^ overload in dystrophic myotubes in our model.

## Figures and Tables

**Table 1:** A list of chemicals used in the Ca^2+^ mobilization assay.

Chemical	Dose	Activity
Tetracaine hydrochloride	50 µM	Voltage-sensitive Ca release inhibitor
MRS1845	10 µM	Potent SOC blocker
2-APB	10 µM	Inhibit Ca^2+^ release from SOC
SKF96365	10 µM	Pan TRPC inhibitor
GsMTx4	1 µM	TRPC1/6 inhibitor
Ruthenium Red	10 µM	Pan TRPV and mitochondrial Ca^2+^ uptake inhibitor
Ryanodine	10 µM	Ca^2+^ release inhibitor from SR via RyR
Nifedipine	10 µM	L-type Ca channel blocker
ML 9	10 µM	SOC inhibitor
YM 58483	10 µM	SOC blocker
Disulfiram	10 µM	Reversibly stimulate Ca-ATPase
Ochratoxin A	10 µM	Stimulate SR ATP-dependent Ca^2+^ pump
Istaroxime	10 µM	Stimulate SERCA2a
5J 4	10 µM	SOC blocker
Cyclopiazonic acid	10 µM	Ca^2+^-ATPase inhibitor
Thapsigargin	10 µM	SERCA inhibitor

SOC, store-operated Ca^2+^ channel; SR, sarcoplasmic reticulum; SERCA, SR Ca^2+^-ATPase.

## Data Availability

This study did not generate/analyze datasets/code.

## Supplementary Materials

The following are available online at https://www.mdpi.com/article/10.3390/biomedicines9111589/s1, Figure S1: gene expression analyses of hiPSC-skeletal myotubes differentiated by the standard replating method, Figure S2: Total Ca^2+^ content of hiPSC-skeletal myotubes, Figure S3: siRNA-mediated Orai1 and STIM1L knockdown on differentiated myotubes by the modified replating, Table S1: A list of primer sequences.
