# Supplementary material for: Orai1–STIM1 Regulates Increased Ca2+ Mobilization, Leading to Contractile Duchenne Muscular Dystrophy Phenotypes in Patient-Derived Induced Pluripotent Stem Cells
Source: Biomedicines. 2021 Oct 31;9(11):1589. doi: 10.3390/biomedicines9111589 (PMC8615222; doi:10.3390/biomedicines9111589)
Supplement: Supplementary file 1 [file biomedicines-09-01589-s001.zip › Supplemental Figure V2.pdf]

S-Figure.1

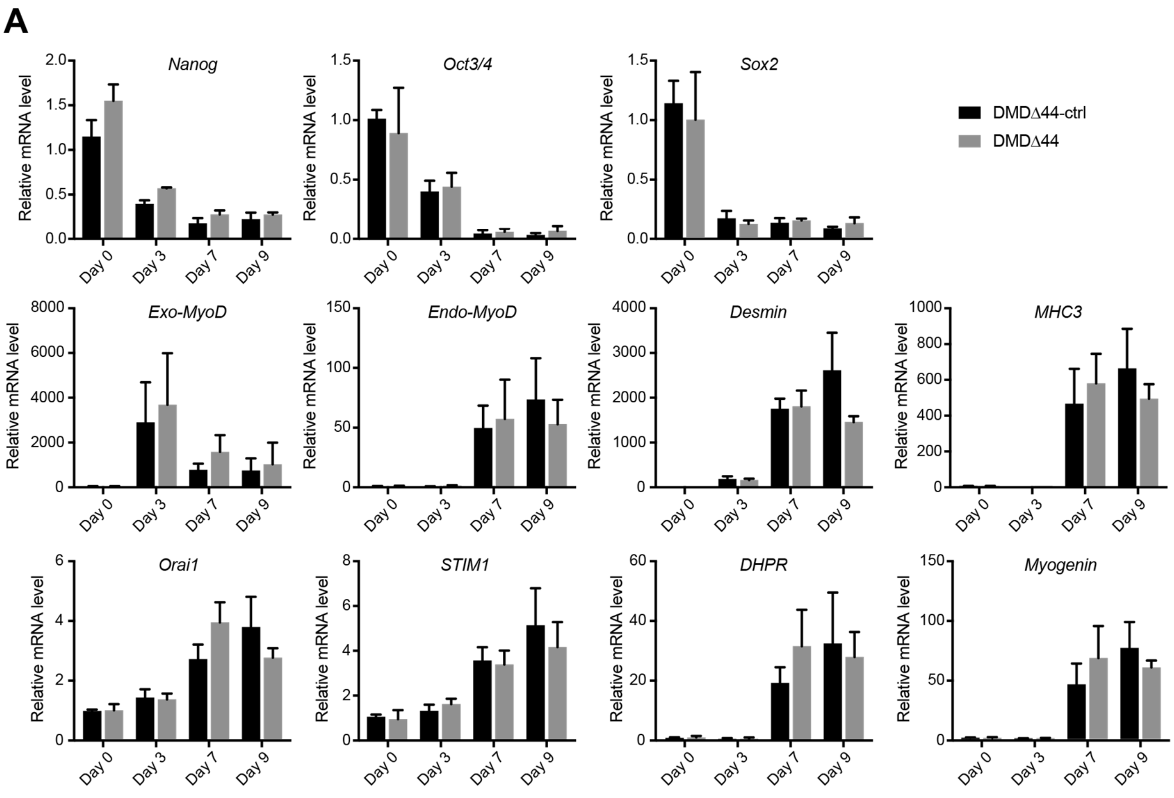

Figure S1. Gene expression analyses of hiPSC-skeletal myotubes differentiated by the standard replating method. (A) RT-qPCR analysis of *Nanog*, *Oct3/4*, *Sox2*, exogenous-*MyoD*, endogenouse-*MyoD*, *Desmin*, *MHC2*, *Orai1*, *STIM1*, *DHPR* and *Myogenin* of DMDΔ44-ctrl and DMDΔ44 myotubes differentiated by the standard replating method. Data represent the mean ± SD and were analyzed with an unpaired *t*-test from three biological replicates. * indicates $P < 0.05$.

S-Figure.2

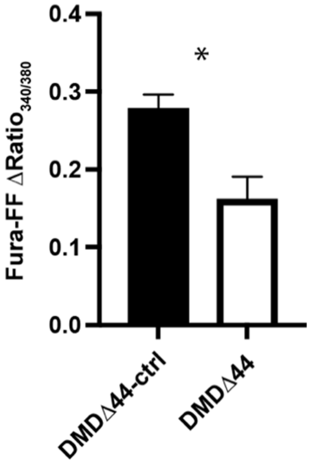

Figure S1. Total releasable Ca^{2+} store content analyses of hiPSC-skeletal myotubes. Data represent the mean ± SD and were analyzed with an unpaired *t*-test from three biological replicates. * indicates $P < 0.05$.

S-Figure.3

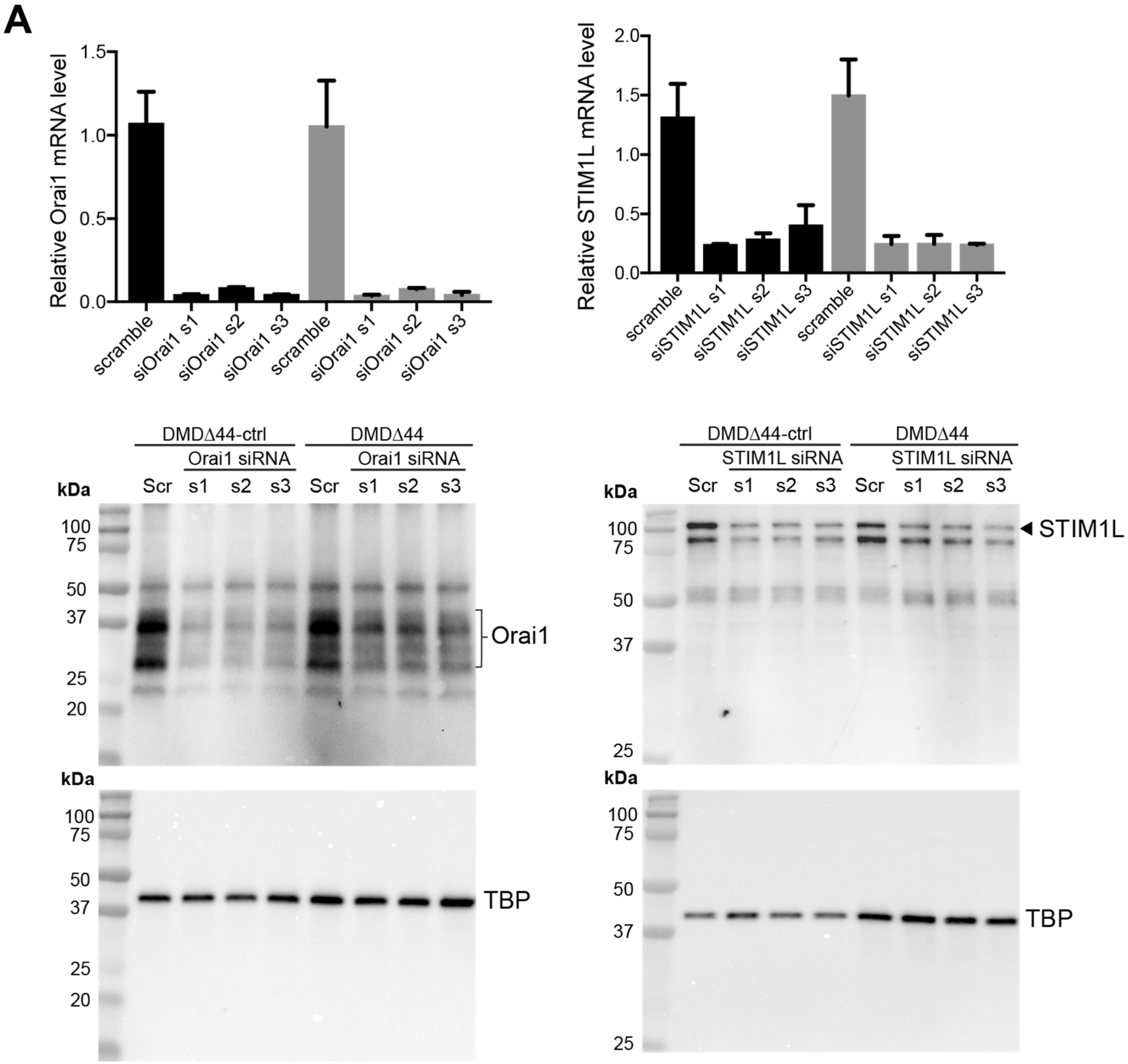

Figure S3. siRNA-mediated Orai1 and STIM1L knock-down on differentiated myotubes by the modified replating. siRNA-mediatead Orai1 and STIM1 knock-down was confirmed by (A) RT-qPCR analysis and (B) Western blot.
