# Supplementary material for: Orai1–STIM1 Regulates Increased Ca2+ Mobilization, Leading to Contractile Duchenne Muscular Dystrophy Phenotypes in Patient-Derived Induced Pluripotent Stem Cells
Source: Biomedicines. 2021 Oct 31;9(11):1589. doi: 10.3390/biomedicines9111589 (PMC8615222; doi:10.3390/biomedicines9111589)
Supplement: Supplementary file 1 [file biomedicines-09-01589-s001.zip › Supplemental Table S1.pdf]

Supplemental Table S1. A list of primer sequences.

Gene	Fwd (5'-3')	Rev (5'-3')
CKM	CATGGCCAAGGTACTGACC	TGATGGGGTCAAAGAGTTCC
Exo-MyoD	CCCCTTCACCATGGAGCTA	AGTGCTCTTCGGGTTTCAGG
Endo-MyoD	CACTCCGGTCCCAAATGTAG	TTCCCTGTAGCACCACACAC
MHC	GCAGATTGAGCTGGAAAAGG	TCAGCTGCTCGATCTCTTCA
Myogenin	TGGGCGTGTAAGGTGTGTAA	CGATGTACTGGATGGCACTG
TBP	GCTGGCCCATAGTGATCTTT	CATCTCCAGCACACTCTTCTC
